# *Streptococcus suis* Serotype 2 Capsule In Vivo

**DOI:** 10.3201/eid2210.151640

**Published:** 2016-10

**Authors:** Jean-Philippe Auger, Nattakan Meekhanon, Masatoshi Okura, Makoto Osaki, Marcelo Gottschalk, Tsutomu Sekizaki, Daisuke Takamatsu

**Affiliations:** Université de Montréal, St-Hyacinthe, Quebec, Canada (J.-P. Auger, M. Gottschalk);; The University of Tokyo, Tokyo, Japan (N. Meekhanon, T. Sekizaki);; Kasetsart University, Bangkok, Thailand (N. Meekhanon);; National Agriculture and Food Research Organization, Tsukuba, Japan (M. Okura, M. Osaki, D. Takamatsu);; Gifu University, Gifu, Japan (D. Takamatsu)

**Keywords:** Streptococcus suis, bacterial capsule, zoonosis, mutation, bacteria, pigs, swine, occupational health, zoonoses, porcine endocarditis

## Abstract

Many *Streptococcus suis* isolates from porcine endocarditis in slaughterhouses have lost their capsule and are considered avirulent. However, we retrieved capsule- and virulence-recovered *S. suis* after in vivo passages of a nonencapsulated strain in mice, suggesting that nonencapsulated *S. suis* are still potentially hazardous for persons in the swine industry.

*Streptococcus suis* is a gram-negative bacterium that infects pigs and causes severe economic losses to the swine industry. Moreover, it causes severe disease in persons in close contact with diseased pigs or their products (*1*). In Japan, *S. suis* has been frequently isolated from pigs with endocarditis in slaughterhouses; most of the isolates were expected to be sequence types (STs) that are potentially hazardous to humans (*2*). Many isolates from porcine endocarditis lost their capsule, and all the nonencapsulated isolates analyzed had mutations in the capsular polysaccharide synthesis (*cps*) genes (*3*,*4*). The capsule of *S. suis* is a major virulence factor (*1*). Although loss of the capsule gives *S. suis* some benefit in causing endocarditis by enhancing the ability of bacterial cells to adhere to porcine and human platelets, a major virulence determinant for infective endocarditis (*3*), nonencapsulated *S. suis* are generally considered avirulent (*5*). However, whether nonencapsulated *S. suis* lurking in porcine endocarditis poses a threat to persons working in the swine industry is unknown. To investigate whether nonencapsulated *S. suis* can restore the ability to express the capsule and become virulent again, we repeated in vitro or in vivo passages of nonencapsulated *S. suis* and attempted to retrieve capsule-recovered strains.

## The Study

For the in vitro passages, we used 29 *S. suis* strains isolated from pigs with endocarditis. These isolates had the *cps* gene cluster of serotype 2 but had lost their capsule because of mutations in the *cps* genes (Table). We subcultured them twice in liquid media and separated the cells according to the buoyant density by Percoll density gradient centrifugation (Technical Appendix). Because encapsulated cells show lower density than nonencapsulated cells (*6*,*7*), we investigated capsular expression of *S. suis* cells with low density by coagglutination tests using serotype 2 antiserum (Technical Appendix). The retrieved *S. suis* was also used for the next subcultures. We repeated 4 cycles of this experiment (in total 8 subcultures) but obtained no encapsulated *S. suis* from any of the strains tested.

**Table:** Nonencapsulated *Streptococcus suis* strains isolated from pigs with endocarditis and used for in vitro passages to investigate possible capsule recovery

Strain	Affected gene(s)	Types of mutations	Affected nucleotide(s) (affected amino acid)	Reference
NL100	*cps2F*	Nonsense	T696G (Tyr232TERM)	(*4*)
NL119	*cps2F*	Missense	T490C (Cys164Arg)	(*4*)
NL122	*cps2F*	Missense	G52A (Gly18Ser)	(*4*)
NL126	*cps2F*	Frameshift by insertion	TCCG	(*4*)
NL132	*cps2E*	Missense	G1199A (Arg400Lys)	(*4*)
	*cps2H*	Frameshift by deletion	TA	(*4*)
NL143	*cps2F*	Missense	G493T (Asp165Tyr)	(*4*)
	*cps2K*	Insertion	AATCATTGG	(*4*)
	*cps2R*	Missense	G496A (Gly166Arg)	(*4*)
NL146	*cps2F*	Nonsense	T482A (Leu162TERM)	(*4*)
NL171	*cps2E*	Insertion	IS element: 1,619 bp	(*3*)
NL174	*cps2H*	Frameshift by deletion	A	(*4*)
NL175	*cps2H*	Frameshift by deletion	A	(*4*)
NL184	*cps2E*	Insertion	IS element: 1,115 bp	(*3*)
NL194	*cps2E*	Insertion	IS element: 1,416 bp	(*3*)
NL208	*cps2E*	Frameshift by deletion	TAAG	(*4*)
NL219	*cps2E*	Frameshift by deletion	TAAG	(*4*)
NL225	*cps2F*	Frameshift by insertion	CCAAA	(*4*)
NL230	*cps2F*	Frameshift by insertion	A	(*4*)
NL240	*cps2E*	Nonsense	C1189T (Gln397TERM)	(*4*)
NL245	*cps2E*	Frameshift by insertion	T	(*4*)
NL249	*cps2E*	Frameshift by insertion	AGCA	(*4*)
NL255	*cps2E*	Insertion	IS element: 1,619 bp	(*3*)
NL257	*cps2E*	Frameshift by insertion	ATCT	(*4*)
NL266	*cps2E*	Frameshift by deletion	A	(*4*)
NL278	*cps2F*	Missense	T259C (Ser87Pro)	(*4*)
NL295	*cps2F*	Missense	T492G (Cys164Trp)	(*4*)
NL303	*cps2F*	Deletion	81 bp	(*4*)
NL322	*cps2B*	Missense	G469A (Asp157Asn)	(*4*)
	*cps2G*	Deletion	50 bp	(*4*)
NL328	*cps2F*	Frameshift by deletion	AG	(*4*)
NL342	*cps2E*	Frameshift by deletion	TAAG	(*4*)
NL345	*cps2H*	Deletion	23 bp	(*4*)
	*cps2N*	Missense	C706T (Pro236Ser)	(*4*)

**Figure 1:**
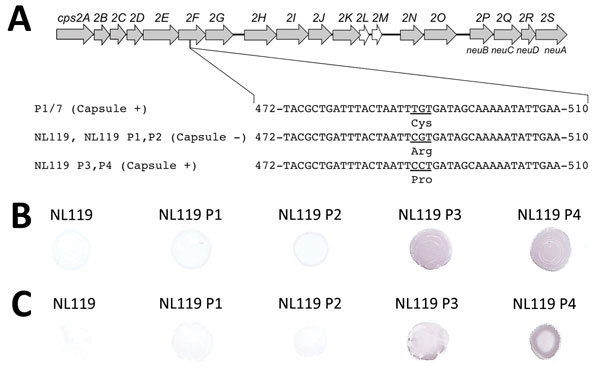
Capsule recovery of *Streptococcus suis* strain NL119 in vivo. A) The genetic organization of the *S. suis* serotype 2 capsular polysaccharide synthesis (*cps*) gene cluster and mutations observed in isolate NL119 and strains retrieved from NL119-infected mice after each in vivo passage (NL119 P1–P4; DDBJ/EMBL/GenBank accession nos. LC147077, LC147078, LC147079, LC147080, and LC077855, respectively). Gray arrows indicate genes putatively involved in capsule synthesis; open arrows indicate genes with unknown functions; numbers indicate nucleotide positions in *cps2F*. NL119 lost the ability to synthesize the capsule because of a missense mutation at nt 490 (T490C, Cys164Arg) of *cps2F* (*4*). B, C) NL119 P1 and P2 retrieved from mice after the first and second in vivo passages, remained nonencapsulated, and their *cps2F* sequences were identical to that of NL119. In NL119 P3 and P4 retrieved after the third and fourth passages, a further missense mutation at nt 491 (G491C, Arg164Pro) of *cps2F* restored the function of the gene, resulting in capsule recovery of the strains. Dot-ELISA of NL119 and strains retrieved from NL119-infected mice after each in vivo passage (NL119 P1–P4) using monoclonal antibody Z3 (B) and polyclonal anti–*S. suis* serotype 2 serum adsorbed with NL119 (C). Monoclonal antibody Z3 specifically recognizes the sialic acid moiety of the *S. suis* serotype 2 capsule.

Although these results suggested that mutations in *cps* genes are not repaired easily, the conditions faced by *S. suis* in vivo could influence capsular expression. To investigate this possibility, we selected strain NL119 as a representative. NL119 is an ST1 strain, one of the types hazardous to humans, but one that has lost the capsule because of a point mutation that occurred at nt 490 (T490C, Cys164Arg) of a glycosyltransferase gene (*cps2F*) (Table; Figure 1, panel A) (*4*). We inoculated groups of 5 mice with 5 × 10^8^ CFU of NL119 (online Technical Appendix). Bacteria persistent in mice were retrieved 36 h after infection from the blood, in which capsular expression works favorably for survival. We investigated capsular expression of the retrieved NL119 by coagglutination tests and used the colony giving the strongest reaction within 30 s for the subsequent in vivo passage.

As expected, the coagglutination test of the parental strain NL119 showed a negative result. Similarly, NL119 after the first and second passages (NL119 P1 and P2, respectively) reacted weakly, comparable to those of the parental strain, suggesting poor encapsulation. Meanwhile, NL119 after the third and fourth passages (NL119 P3 and P4, respectively) reacted strongly, suggesting recovery of the capsule. To confirm this finding, we further analyzed formalin-killed bacteria by dot-ELISA using monoclonal antibody Z3, which reacts with the sialic acid moiety of the serotype 2 capsule (*8*), and an anti–*S. suis* serotype 2 serum adsorbed with parental strain NL119 to select the capsule-specific antibodies (Technical Appendix). In accordance with the coagglutination test, NL119 P1 and P2 gave weak reactions similar to those of NL119, whereas strong signals were detected in NL119 P3 and P4 with both the monoclonal antibody and serum (Figure 1, panels B, C). Because NL119 P1–P4 were also ST1 as determined by multilocus sequence typing, these results suggested that NL119 had recovered the capsule during passages in animals.

To find mutations that had contributed to the capsule recovery, we sequenced the *cps2F* gene of NL119 P1–P4. Although the cytosine residue at nt 490 was not changed in comparison with the parental strain, we found a further missense mutation at nt 491 (G491C, Arg164Pro) of the *cps2F* gene in NL119 P3 and P4 (Figure 1, panel A). To investigate whether this mutation was involved in the capsule recovery, we cloned *cps2F* of NL119 P4 into a gene expression vector pMX1 (*9*) and introduced it into the parental strain NL119 (Technical Appendix). A coagglutination test using serotype 2 antiserum showed positive reactions in all transformants tested, demonstrating that the further missense mutation restored the function of *cps2F*, resulting in capsule recovery of NL119 P3 and P4, although how the Cps2F function was recovered by the amino acid substitution is unknown. Isolation of the capsule-recovered strains in vivo could have been the consequence of selection of encapsulated cells, which were already present as a subpopulation in the original nonencapsulated NL119 population, by resisting host immunity including phagocytosis. However, because NL119 was a nonencapsulated strain originally recovered from a single colony and well-isolated by repeated passages in vitro, and no encapsulated subpopulation was ever retrieved in vitro by the selection using Percoll density gradient centrifugation, the most plausible hypothesis would be that the capsule-recovered *S. suis* was generated in vivo.

**Figure 2:**
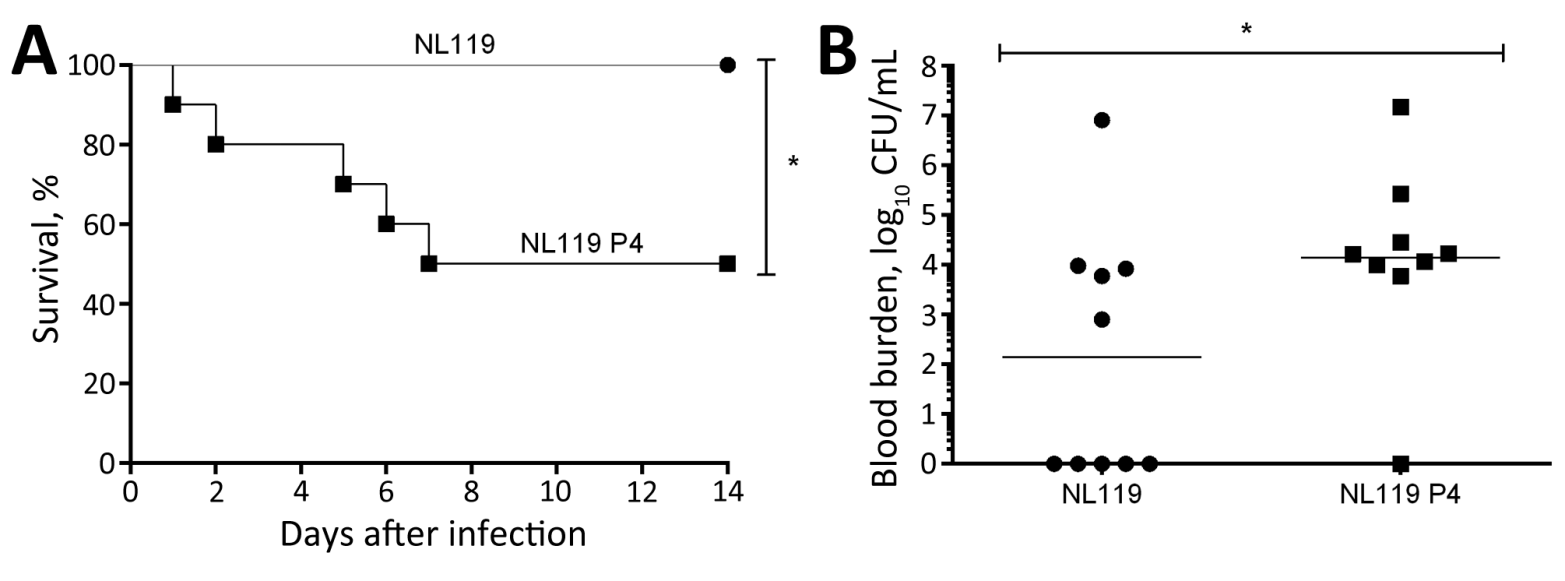
Virulence of nonencapsulated *Streptococcus suis* strain NL119 and capsule-recovered NL119 P4 in mice. A) Survival of C57BL/6 mice (n = 10 mice per strain; until 14 days after infection) inoculated intraperitoneally with 5 × 10^7^ CFU of either NL119 or NL119 P4. B) Blood bacterial burden at 24 h after infection. Data of individual mice are presented as log_10_ CFU/mL with the geometric mean. Asterisks indicate a significant difference between NL119 and NL119 P4 (p<0.05).

To evaluate whether the capsule-recovered *S. suis* isolate also recovered its virulence, we infected mice with either NL119 or NL119 P4 (online Technical Appendix). Rates of death differed significantly (p<0.05): 50% death in the NL119 P4-infected mice 14 days after infection, compared with 0% for the nonencapsulated NL119 (Figure 2, panel A). Recovery of the capsule also significantly increased its survival in blood 24 h after infection (p<0.05). All but 1 surviving NL119 P4-infected mice had significant blood bacterial titers (>5 × 10^3^ CFU/mL; geometric mean 10^4^ CFU/mL). In contrast, except for 1 mouse, all mice infected with NL119 had blood bacterial titers <10^4^ CFU/mL (geometric mean 10^2^ CFU/mL) (Figure 2, panel B).

## Conclusions

Although capsule loss might contribute to *S. suis* infection by enhancing bacterial adherence to host cells and biofilm formation (*3*,*10*–*12*), capsule loss makes *S. suis* cells susceptible to phagocytosis; therefore, the virulence of nonencapsulated mutants was attenuated when evaluated in animal models (*5*). In accordance with previous studies, nonencapsulated NL119 was avirulent. However, NL119 P4, which recovered its capsule in vivo, also recovered virulence. Because various mutations in *cps* genes, including large deletions and insertions, cause capsule loss in *S. suis* (*3*,*4*), not all mutations will be repaired like NL119. However, our results demonstrated the presence of a nonencapsulated mutant, which can recover the capsule and virulence in vivo. Hence, nonencapsulated *S. suis* strains can cause severe diseases to the next hosts by recovering the capsule, which indicates that some nonencapsulated *S. suis* lurking in pigs with endocarditis are still potentially hazardous to persons handling such pigs and their products. Further investigations using a variety of naturally occurring and laboratory-derived mutants are needed for a comprehensive understanding of the biological significance and mechanisms of this phenomenon.

Technical Appendix Expanded methods for study of possible capsule recovery of nonencapsulated *Streptococcus suis*.
