# Correction: Vol. 22, No. 10

**DOI:** 10.3201/eid2308.C12308

**Published:** 2017-08

**Keywords:** errata, erratum, correction

*Streptococcus suis* was incorrectly described in the text of *Streptococcus suis* Serotype 2 Capsule In Vivo (Auger J et al.). It is a gram-positive bacterium. The article has been corrected online (https://wwwnc.cdc.gov/eid/article/22/10/15-1640_article).

