# Supplementary material for: Streptococcus suis Serotype 2 Capsule In Vivo
Source: Emerg Infect Dis. 2016 Oct;22(10):1793–6. doi: 10.3201/eid2210.151640 (PMC5038428; doi:10.3201/eid2210.151640)
Supplement: Technical Appendix — Expanded methods for study of possible capsule recovery of nonencapsulated Streptococcus suis. [file 15-1640-Techapp-s1.pdf]

Article DOI: http://dx.doi.org/10.3201/eid2210.151640

Streptococcus suis Serotype 2 Capsule In Vivo

Technical Appendix

Materials and Methods

In vitro Passages of Nonencapsulated *Streptococcus suis* Strains in Culture Media and Selection of *S. suis* Cells with Low Buoyant Density

Nonencapsulated *S. suis* strains isolated from heart valve vegetations of pigs with endocarditis (*1*,*2*) were cultured overnight in 5 mL of Todd-Hewitt broth (THB; Becton Dickinson, Sparks, MD, USA) or THB containing 2% glucose and 1.5% disodium phosphate (THGB) at 37°C (first subculture). A total of 150 μL culture was then inoculated into 5 mL of fresh THB or THGB and cultured at 37°C for 9–12 h (second subculture). The bacterial cells subcultured twice were collected by centrifugation at 3,500 rpm for 20 min and washed once with 1 mL of 0.15 mol/L NaCl. Because encapsulated bacterial cells show lower buoyant density than nonencapsulated cells (*3*,*4*), the washed *S. suis* cells were then separated according to the buoyant density by Percoll density gradient centrifugation as follows.

For preparation of a stock isotonic Percoll (SIP) solution, Percoll PLUS (GE Healthcare UK Ltd, Buckinghamshire, UK) was diluted with 1.5 mol/L NaCl in a ratio of 9:1, and the pH was adjusted to 7.0 with 1 mol/L HCl. The subcultured and washed *S. suis* cells were suspended with 100 μL of the undiluted Percoll and added to the bottom of a 15-mL centrifuge tube. Solutions of 20%, 40%, 60%, and 80% SIP were prepared by further dilution of the SIP with 0.15 mol/L NaCl. Two milliliters each of these solutions was gently layered onto the *S. suis* suspension in the tube to produce a step gradient with 80% SIP at the bottom and 20% SIP at the top. The tube was centrifuged at 2,600 × gravity for 20 min to separate bacterial cells according to the density.

After the centrifugation, most of the *S. suis* cells, which were considered to be still nonencapsulated, were concentrated at the interface between 60% and 80% SIP. To retrieve *S. suis* cells with a lower density (i.e., *S. suis* cells that might express the capsule), we removed 50 μL of

the solution from the interface between 20% and 40% SIP. The removed solution was spread on Todd-Hewitt agar (THA) plates and cultured overnight at 37°C in air plus 5% CO_2. The capsular expression of colonies grown on the plates was evaluated by coagglutination tests using the serotype 2 antiserum (*1*). Five to 10 colonies on the THA plates were also used as an inoculum for the next 2 times subcultures, and capsular expression of the subcultured bacteria was evaluated again after Percoll density gradient centrifugation. We repeated this experiment 4 times for each strain (in total 8 subcultures for each strain).

Animal Ethics

Animal experiments were performed at the Université de Montréal (St-Hyacinthe, QC, Canada). All the experiments were conducted in strict accordance with the recommendations and approved by the Université de Montréal Animal Welfare Committee guidelines and policies.

In vivo Passages of Nonencapsulated *S. suis* in Mice

For in vivo passages, a well-established C57BL/6 mouse model of infection (*5*) was used. Nonencapsulated *S. suis* NL119 was passaged a total of 4 times in mice. For preparation of inocula, the strain was grown overnight on Columbia Agar supplemented with 5% sheep blood (Oxoid, Nepean, ON, Canada) at 37°C with 5% CO_2. Five milliliters of THB was inoculated with NL119 grown on the agar and incubated for 8 h at 37°C with 5% CO_2. Working cultures were prepared by inoculating 30 mL of THB with 10 μL of a 10^{-3} dilution of the 8 h cultures and incubating for 16 h at 37°C with 5% CO_2. Bacteria were washed twice with pH 7.3 phosphate-buffered saline, suspended in THB, appropriately diluted, and plated on THA to accurately determine bacterial concentrations.

Six- to 10-week-old C57BL/6 mice (Jackson Laboratory, Bar Harbor, ME, USA) were acclimatized to standard laboratory conditions with unlimited access to water and rodent chow. The mice were then inoculated with 5×10^8 CFU of NL119 by intraperitoneal injection, and bacteria were isolated from the blood of the surviving mice 36 h after infection by collecting 5 μL blood from the tail vein and plating the proper dilutions on THA. Five mice were used for each of the 4 passages (20 mice total). After each in vivo passage, 5 colonies from each surviving mouse were individually inoculated in 5 mL of THB and incubated for 16 h at 37°C with 5% CO_2. After incubation, bacteria were collected by centrifugation for 10 min at 3,300 × gravity and suspended in phosphate-buffered saline with 0.5% formalin. The capsular expression of these isolates was

evaluated by coagglutination tests (*6*). The colony giving the strongest reaction within 30 sec was used for the subsequent in vivo passage. Capsular expression of strains selected by coagglutination after each passage was also confirmed by dot-ELISA as previously described, using both monoclonal antibody Z3 supernatant, which reacts with the sialic acid moiety of the *S. suis* serotype 2 capsule, and polyclonal anti–*S. suis* serotype 2 rabbit serum (*7*,*8*). To selectively obtain antibodies against the capsule, rabbit serum was adsorbed with an overnight culture of strain NL119 for 1 h at 37°C with agitation, for a total of 6 cycles (*8*).

Multilocus Sequence Typing

Multtilocus sequence typing (MLST) was performed by sequencing 7 housekeeping genes, as described previously (*9*). The sequence types of NL119 P1-P4 were determined by comparing the determined sequences with those in the *S. suis* MLST database (http://ssuis.mlst.net).

Sequencing of *cps2F*

Genomic DNA of *S. suis* NL119 and NL119 P1-P4 was extracted as described previously (*10*). The entire *cps2F* gene was amplified by KOD FX (TOYOBO Co., Ltd, Osaka, Japan) and Ex Taq DNA polymerase (Takara Bio Inc., Kusatsu, Japan) with primers cps2-F6 and cps2-R6 (*1*) and directly sequenced by a BigDye Terminator v3.1 cycle sequencing kit using 3130*xl* Genetic Analyzer (Applied Biosystems, Tokyo, Japan). SEQUENCHER Ver. 5.2 (Gene Codes Corp., Ann Arbor, MI, USA) was used for assembly and analysis of the sequences.

Construction of NL119 P4-Derived *cps2F* Expression Vector and Complementation Analysis

To construct the NL119 P4-derived *cps2F* expression vector, the appropriate gene region was amplified from the genomic DNA of strain NL119 P4 by PCR with the primers CPS2F-F3 (5′-TTGAGGATCCAGGGAAGTAAGTAAGACTCC-3′) and CPS2F-R (5′-TGCCCCATAGAATTCTGCTCCAGCATGGAG-3′), digested by *Bam*HI and *Eco*RI, and cloned into the respective sites of pMX1 (*11*). After being introduced into *Escherichia coli* strain MC1061 (*12*), the direction and sequences of the cloned genes were verified by PCR and sequencing. The resultant expression vector was then introduced into nonencapsulated *S. suis* NL119 by the method described previously (*13*), and the restoration of capsular expression was examined by coagglutination tests.

Experimental Infection of Mice to Investigate the Virulence of NL119 and NL119 P4

To compare the virulence of nonencapsulated and capsule-recovered strains, 5×10^7 CFU of NL119 or NL119 P4 was administered to 6-week-old C57BL/6 mice (10 mice/group) by intraperitoneal injection. Mice were monitored at least 3 times daily until 72 h after infection and then twice daily until 14 days after infection for clinical signs and death. Bacteremia of the surviving mice was evaluated 24 h after infection by using blood collected from the tail vein. An unpaired *t* test and log-rank (Mantel-Cox) test were performed to compare blood bacterial burden and survival, respectively. $p<0.05$ was considered as the threshold for statistical significance.

References

1. Lakkitjaroen N, Takamatsu D, Okura M, Sato M, Osaki M, Sekizaki T. Loss of capsule among *Streptococcus suis* isolates from porcine endocarditis and its biological significance. J Med Microbiol. 2011;60:1669–76. PubMed http://dx.doi.org/10.1099/jmm.0.034686-0

2. Lakkitjaroen N, Takamatsu D, Okura M, Sato M, Osaki M, Sekizaki T. Capsule loss or death: the position of mutations among capsule genes sways the destiny of *Streptococcus suis*. FEMS Microbiol Lett. 2014;354:46–54. PubMed http://dx.doi.org/10.1111/1574-6968.12428

3. Håkansson S, Bergholm A-M, Holm SE, Wagner B, Wagner M. Properties of high and low density subpopulations of group B streptococci: enhanced virulence of the low density variant. Microb Pathog. 1988;5:345–55. PubMed http://dx.doi.org/10.1016/0882-4010(88)90035-6

4. Patrick S, Reid JH. Separation of capsulate and non-capsulate *Bacteroides fragilis* on a discontinuous density gradient. J Med Microbiol. 1983;16:239–41. PubMed http://dx.doi.org/10.1099/00222615-16-2-239

5. Domínguez-Punaro ML, Segura M, Radzioch D, Rivest S, Gottschalk M. Comparison of the susceptibilities of C57BL/6 and A/J mouse strains to *Streptococcus suis* serotype 2 infection. Infect Immun. 2008;76:3901–10. PubMed http://dx.doi.org/10.1128/IAI.00350-08

6. Gottschalk M, Higgins R, Boudreau M. Use of polyvalent coagglutination reagents for serotyping of *Streptococcus suis*. J Clin Microbiol. 1993;31:2192–4. PubMed

7. Charland N, Jacques M, Lacouture S, Gottschalk M. Characterization and protective activity of a monoclonal antibody against a capsular epitope shared by *Streptococcus suis* serotypes 1, 2 and 1/2. Microbiology. 1997;143:3607–14. PubMed http://dx.doi.org/10.1099/00221287-143-11-3607

8. Lecours M-P, Fittipaldi N, Takamatsu D, Okura M, Segura M, Goyette-Desjardins G, et al. Sialylation of *Streptococcus suis* serotype 2 is essential for capsule expression but is not responsible for the main capsular epitope. Microbes Infect. 2012;14:941–50. PubMed http://dx.doi.org/10.1016/j.micinf.2012.03.008

9. King SJ, Leigh JA, Heath PJ, Luque I, Tarradas C, Dowson CG, et al. Development of a multilocus sequence typing scheme for the pig pathogen *Streptococcus suis*: identification of virulent clones and potential capsular serotype exchange. J Clin Microbiol. 2002;40:3671–80. PubMed http://dx.doi.org/10.1128/JCM.40.10.3671-3680.2002

10. Wilson K. Preparation of genomic DNA from bacteria. Curr Protoc Mol Biol. 2001 Nov; Chapter 2:Unit 2.4. http://dx.doi.org/10.1002/0471142727.mb0204s56 PubMed

11. Okura M, Osaki M, Fittipaldi N, Gottschalk M, Sekizaki T, Takamatsu D. The minor pilin subunit Sgp2 is necessary for assembly of the pilus encoded by the *srtG* cluster of *Streptococcus suis*. J Bacteriol. 2011;193:822–31. PubMed http://dx.doi.org/10.1128/JB.01555-09

12. Casadaban MJ, Cohen SN. Analysis of gene control signals by DNA fusion and cloning in *Escherichia coli*. J Mol Biol. 1980;138:179–207. PubMed http://dx.doi.org/10.1016/0022-2836(80)90283-1

13. Zaccaria E, van Baarlen P, de Greeff A, Morrison DA, Smith H, Wells JM. Control of competence for DNA transformation in *Streptococcus suis* by genetically transferable pherotypes. PLoS One. 2014;9:e99394. PubMed http://dx.doi.org/10.1371/journal.pone.0099394
